# Life science experiments performed in space in the ISS/Kibo facility and future research plans

**DOI:** 10.1093/jrr/rrw020

**Published:** 2016-08-16

**Authors:** Takeo Ohnishi

**Affiliations:** Department of Radiation Oncology, School of Medicine, Nara Medical University, 840 Shijo-cho, Kashihara, Nara 634–8521, Japan

**Keywords:** space radiation, gene expression, DNA damage, adaptive response, heavy particles

## Abstract

Over the past several years, current techniques in molecular biology have been used to perform experiments in space, focusing on the nature and effects of space radiation. In the Japanese ‘Kibo’ facility in the International Space Station (ISS), the Japan Aerospace Exploration Agency (JAXA) has performed five life science experiments since 2009, and two additional experiments are currently in progress. The first life science experiment in space was the ‘Rad Gene’ project, which utilized two human cultured lymphoblastoid cell lines containing a mutated ***p53*** gene (m***p53***) and a parental wild-type ***p53*** gene (wt***p53***) respectively. Four parameters were examined: (i) detecting space radiation–induced DSBs by observing γH2AX foci; (ii) observing ***p53***-dependent gene expression during space flight; (iii) observing ***p53***-dependent gene expression after space flight; and (iv) observing the adaptive response in the two cell lines containing the mutated and wild type ***p53*** genes after exposure to space radiation. These observations were completed and have been reported, and this paper is a review of these experiments. In addition, recent new information from space-based experiments involving radiation biology is presented here. These experiments involve human cultured cells, silkworm eggs, mouse embryonic stem cells and mouse eggs in various experiments designed by other principal investigators in the ISS/Kibo. The progress of Japanese science groups involved in these space experiments together with JAXA are also discussed here. The Japanese Society for Biological Sciences in Space (JSBSS), the Utilization Committee of Space Environment Science (UCSES) and the Science Council of Japan (ACJ) have supported these new projects and new experimental facilities in ISS/Kibo. Currently, these organizations are proposing new experiments for the ISS through 2024.

## INTRODUCTION

Two specific factors in the space environment, microgravity and space radiation, are very significant concerns for humans travelling in space. It is expected that the biological data obtained from space-based experiments will be of great utility in designing systems for the physiological protection of astronauts from the deleterious effects of space radiation exposures during long stays in space. Space radiation includes many types of radiation that arise from the solar wind, supernovas, and other sources in the galaxy, and these radiation sources produce low-dose rates ranging from 0.5–1.0 mSv per day, along with low doses of <100 mSv, even for long stays of about half a year on the ISS. All Japanese space experiments were monitored with Passive Physical Dosimeters (PADLES) in space to obtain physical dosimetry values. These measurements were obtained by Dr Akiko Nagamatsu *et al*. in the JAXA group for life science experiments [1]. The plastic nuclear detector CR-39 was used to measure high linear-energy-transfer (LET) radiation, and thermo-luminescence dosimeters were used to measure low-LET radiation. In recent flights, physical dosimetry measurements were obtained for Japanese space crews who stayed on the ISS for extended periods of about half a year. This group also obtained dosimetry measurements for other Asian astronauts numerous times. In radiation space experiments, the observed biological effects of space radiation must be compared with concurrent physical dosimetry measurements in order to accurately determine the effects of space radiation. Physical dosimetry monitors were also attached to experimental biological samples and placed in several locations in the ISS/Kibo facility. This provided physical dosimetry information for space radiation on the ISS [1]. It was calculated that the dose rate during recent space flights was about 0.5 mSv per day. The effects of space radiation were also examined in cultured human cells that were kept on the ISS in a frozen state, and this provided biological dosimetry. From these results, the relative biological effectiveness of space radiation was examined, and this information was used to compare results obtained with physical dosimetry and biological dosimetry. Over the past several years, current techniques in molecular biology have also been used in experiments in space to focus on the nature and effects of space radiation in the ISS/Kibo facility. The ISS/Kibo facility contains a freezer that can be used to store human cultured cells, and a CO_2_ incubator with a centrifuge which permits the growth of cells on the ISS at 1 gravity (1 *g*) and at ambient microgravity (µ*g)* levels. Radiation biologists have been utilizing these facilities to design experiments to study the biological effects of space radiation.

## REPORT ON THE RAD GENE PROJECT

**Fig. 1.**
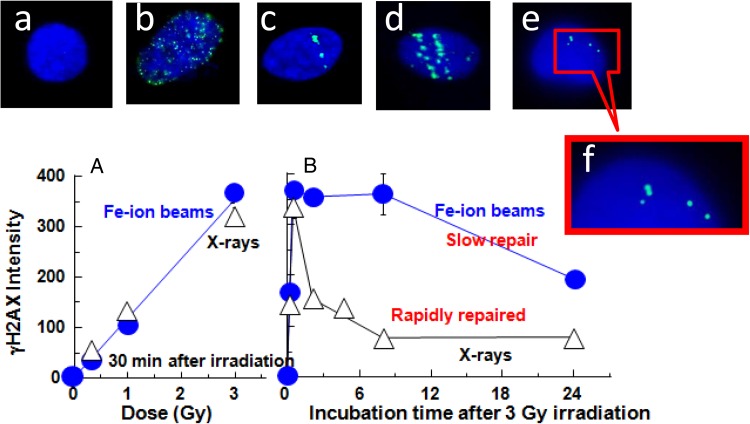
DSB formation and repair. (**A** and **B**) human cultured cells were irradiated with X-rays and Fe-ion (200-KeV/µm) beams on the ground, and then incubated for the indicated periods. (**A**) dose dependency after a 30 min incubation after irradiation. (**B**) Repair efficiency after irradiation with 3 Gy. Photos in the upper panels: DSBs and nuclei were stained by γH2AX-antibody and detected as γH2AX foci (green) and nuclei were stained with DAPI (blue). Typical photographs of each cell are shown in **a**–**f**. (**a**) unirradiated controls; (**b**) X-rays (3 Gy); (**c**) Fe-ion (0.3 Gy); (**d**) Fe-ion (0.6 Gy); (**e**) flight sample in the ISS for 133 days; (**f**) enlarged photograph of ‘**e**’. This figure is derived from figures published in Takahashi *et al*. (2008) and Ohnishi *et al*. (2009), details therein [2, 4].

To accurately interpret the results of space experiments, it is necessary to design careful and comprehensive control experiments on the ground [2]. Cultured normal fibroblast AG1522 cells were used for cell biology experiments. Cells were irradiated with X-rays and Fe-ion beams with low and high linear-energy-transfer values, respectively. Figure 1A shows the presence of DNA double-strand breaks (DSBs), which were detected as γH2AX-positive foci in cell nuclei at 30 min after irradiation. The efficiency of DNA DSB formation was dose-dependent and linear, and almost the same with both types of radiation (Fig. 1A). Observing the pattern of DSB formation, X-rays appear to induce randomly scattered DSB foci in the nucleus (Fig. 1B). In contrast, Fe-ion beams induced DSBs along single linear tracks with multiple foci (Fig. 1C and D). DSB repair was also measured during long incubations after irradiation (Fig. 1B). DSBs induced by X-rays were rapidly repaired, but DSBs induced by Fe-ion beams were repaired slowly. This indicates that the DNA lesions induced by the two types of radiation are repaired differently. It is thought that DSBs induced by Fe-ion beams may be more complex or are processed differently during repair when compared with X-ray–induced DSBs.

The first life science experiment in space was the Rad Gene project, which used two human cultured lymphoblastoid cell lines: WTK1 and TSCE5. The WTK1 cell line harbors a mutated *p53* gene (m*p53*), and the TSCE5 cell line harbors a wild-type *p53* gene (wt*p53*) [3]. Four parameters were examined: (i) detection of space radiation-induced DSBs; (ii) gene expression during space flight; (iii) gene expression after space flight; and (iv) the adaptive response induced by space radiation.

### Detection of DSBs induced by space radiation

WTK1 and TSCE5 cells were transported to the ISS in a frozen state for a 133-day stay in the facility's freezer**.** After being maintained in a freezer during the duration of their space flight, the cells were returned to the ground and were cultured for 30 min in a CO_2_ incubator at 37°C. They were then fixed and stained to examine γH2AX foci. The cellular γH2AX foci were observed along linear tracks (Fig. 1E and F) [4]. This indicated that heavy-ion particles, which are one component of space radiations, induced linear tracks in the nuclei. From the observed frequencies of γH2AX-positive foci, it was calculated that the space radiation dose acquired was 94.5 mSv during 133 days. This was the value determined with biological dosimetry, and the calculated dose rate was ∼0.7 mSv per day. This value was quite similar to the physical dosimetry value, which was 71.2 mSv over 133 days, or 0.5 mSv/day.

### Gene expression during space flight

**Fig. 2.**
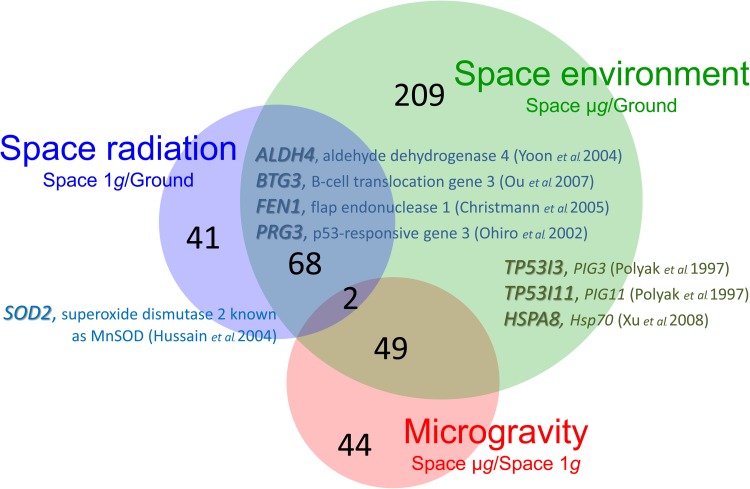
*p53*-Dependent induced genes in cells cultured in the ISS. Values for induced genes were subtracted from values observed in wild-type cells and in m*p53* cells. The large black numbers in the circles are the number of *p53*-dependent induced genes in each situation. (**A**) Space environment (green circle, Space μ*g*/Ground): the induced values in the cultured cells under μ*g* conditions in the ISS were subtracted from the ground control cells. (**B**) Microgravity (pink circle, Space μ*g*/Space 1 *g*): the values for the cultured cells under μ*g* conditions in the ISS were subtracted from the values for cultured cells grown under 1 *g* conditions in the ISS. (**C**) Space radiation (purple circle, Space 1 *g*/Ground). The values for cultured cells under 1 *g* conditions in the ISS were subtracted from the values for cultured cells grown on the ground. This figure is derived from a figure published in Takahashi *et al*. (2010), details therein [5].

The biological effects of microgravity and space radiation on gene and protein expression were examined in *p53*-dependent regulated genes. On the ISS, cell cultures in specially constructed multicompartmented culture bags were transferred from the freezer to a CO_2_ incubator, and the cells were grown in the ISS/Kibo facility. The culture bags were kept in an incubator at 37°C in 1 *g* and in µ*g* conditions for 8 days. Internal partitions in the culture bags were broken, and the WTK1 or TSCE5 cells in one compartment were mixed with stored solutions in the other compartment and then rapidly transferred into a freezer. The mixing procedure between the culture medium and the stored solutions in the second compartment was confirmed by observing the distribution of different colored beads in the two compartments. The cells then remained frozen until they were returned to the ground. On the ground, the control cells were handled with exactly the same protocol as the flight samples. After the flight, RNA was extracted and cDNA was analyzed on DNA arrays. A number of genes were observed with this protocol, and some, for the first time, were reported to be *p53*-regulated genes or involved in *p53* signaling pathways [4]. The space samples were exposed to space radiation and acquired a dose of ∼52 mSv while in a frozen state for 97 days. During culture for 8 days, the samples were exposed to ∼4 mSv, and the total dose was 56 mSv. The effects of microgravity, space radiation and the space environment were compared. The results show the presence of *p53*-dependent enhanced gene expression in the cells cultured in the ISS (Fig. 2). There were 413 genes with altered expression levels among the 41 000 genes observed in these experiments (∼1%). The behavior of several genes was found to correspond well with reported results, and these genes are shown in Fig. 2. These experimental space results have been previously described in detail [3]. However, the activity of other genes that had altered expression levels after exposure to radiation was seen for the first time in these experiments. The behavior of these newly observed induced genes has been reported [4]. The behavior of 68 genes in space was examined, and it was found that microgravity synergistically enhanced the effects of space radiation. In contrast, microgravity effects were not synergistically enhanced by space radiation among 49 other genes observed in a microgravity and space environment. There were 358 genes with altered expression levels that were *p53*-dependent downregulated genes (data not shown) [4]. Protein as well as RNA synthesis was also analyzed in the space samples cultured during space flight in order to observe any *p53*-dependent induction and depression (data not shown). These experimental results have been reported elsewhere [5].

### Gene expression after space flight

It was previously reported that P53 protein accumulated in the muscle of rats after spaceflight. Since P53 proteins are induced by many types of stress, including radiation and heat, this response was utilized in the Rad Gene project [3]. The human WTK1 and TSCE5 cultured cells were kept in a frozen state for 133 days in the ISS/Kibo facility. After flight, the samples were cultured for 6 h at 37^o^C; the RNA was then extracted and the gene expression was analyzed [6]. Gene expression profiles induced by space radiation showed that there were 50 *p53-*dependent upregulated genes and 94 downregulated genes. The total number of genes affected was observed to be 144 genes. Although prominent *p53*-regulated genes were not detected in this experiment, newly identified *p53*-dependent genes were identified. Future studies are now recommended for these newly identified genes and their functions. Although there were many induced genes, the focus was on stress proteins such as *HSPA6*, *HSPA1A*, *HSP90AA1*, *HSP90AB1* and *HSP90AB3P*. These heat shock proteins (HSPs) may also play a role in protecting cellular proteins. Like p53, HSPs are important key proteins involved in a cell's fate. These proteins are probably induced by space radiation, even at low doses such as 76 mSv over 133 days. From the analysis of protein analysis of the flight samples, we also detected these HSPs [7]. Many reports have already been published concerning radiation-induced HSPs [6]. Notably, there is an accumulation of Hsp70 in muscle, skin and spleen of space-exposed goldfish [1]. One of the stress-response activators of p300 stimulates the transcription of *HSP* genes through p53 activity. This data concurred well with these recent reports. Since the samples were frozen, microgravity effects were not relevant here. Three of the downregulated genes, CD44, TNFA1P and TNFRS17, were next studied. These gene products are membrane proteins, and a recent report indicated that p53 depressed *CD44* expression under cell stress conditions. Although the depression of *TNF* activity after spaceflight has been reported, the relationship between *p53* gene status and the gene expression of *TNF* is still unknown. In summary, large differences were found during and after spaceflight in gene expression in response to space radiation. These differences might have arisen from the total exposure dose because DNA damage regulates DNA repair, cell cycle progress, etc. For the four genes downregulated here, the relationship of space radiation exposures and post-flight responses are not yet understood. Other experimental results have been reported elsewhere [6].

### Adaptive response induced by space radiation

**Fig. 3.**
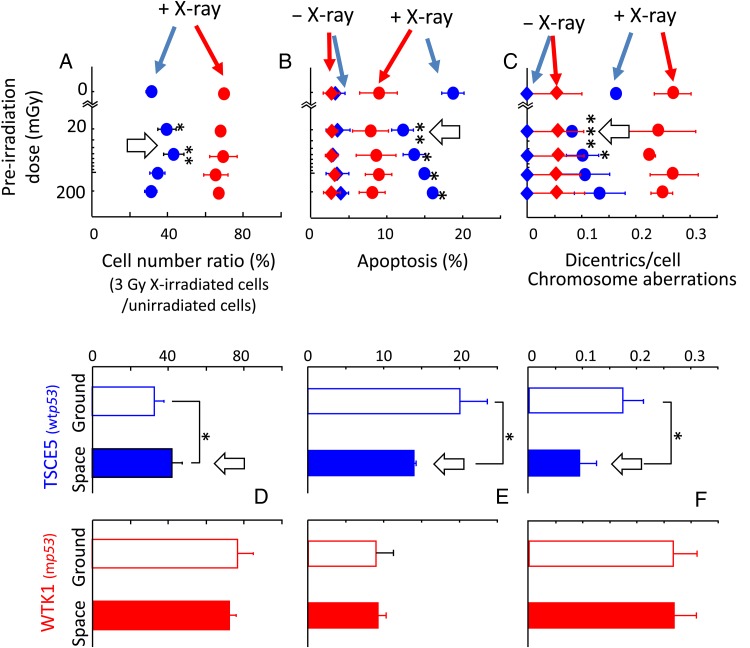
*p53*-Dependent adaptive response in human cells exposed to space radiation. (**A**–**C**) ground experiments. Red circles, WTK1 cells (m*p53*); blue circles, TSCE5 cells (wt*p53*). + X-ray: pre-irradiation followed by a challenging irradiation with 3 Gy; – X-ray: challenging irradiation without a conditioning or pre-irradiation. (**D**–**F**) Space samples (solid columns) and the ground control (open columns) experiments. Cells were kept in a freezer in the ISS for 133 days. After space flight, the cells were cultured for 6 h and then irradiated with a challenging dose of 3 Gy. Thereafter, the cells were analyzed for indicators of the adaptive response [8]. Red columns, WTK1 (m*p53*); blue columns, TSCE5 (wt*p53*). Open white arrows, adaptive response. Error bars indicate standard deviations. *P < 0.05, ***P < 0.01 (Student's *t* test). This figure is derived from a figure published in Takahashi *et al*. (2010), details therein [8].

Reports describing ground control experiments with the radio-adaptive response have appeared. The adaptive response has a specific window or interval between the pre-irradiation conditioning dose and the challenging irradiation, and the response window intervals (with a pre-irradiation dose ranging from 20–100 mGy) are shown in Fig. 3 for *p53* wild-type cells, but not for m*p53* cells. The window depends on the priming dose range (from 20–100 mGy) and on *p53*-dependence. To confirm exposures to space radiation in a frozen state in the ISS, the adaptive response was used as an indicator by looking at cell killing, apoptosis and chromosome aberrations containing dicentrics in WTK1 and TSCE5 cells. The flight samples were cultured on return to the ground and then irradiated with a high dose of X-rays (3 Gy), which served as a challenging irradiation. In the space sample that endured 133 days in a frozen state (Fig. 3D–F), the radio-adaptive response was found only in wt*p53* cells (TSCE5), but not in m*p53* cells (WTK1). These results confirmed that the space radiation dose was in the range of 20–100 mGy during this space flight. Physical dosimetry detected a 94.5 mSv dose during 133 days in the ISS (Fig. 1).

## OTHER JAPANESE PROJECTS IN THE ISS

### Detection of changes in LOH profiles of TK mutants in human cultured cells

Principal investigator (PI): Fumio Yatagai. The purpose of this study was to observe any direct effects from space-radiation exposure under two types of gravity, µ*g* and 1 *g,* during cell culture in the ISS by analyzing TK mutations (LOH analysis). The lymphoblastoid cell lines WTK1 and TSCE5 were used. These are the same cell lines used in the Rad Gene project [9]. This ISS experiment was performed at the same time as the Rad gene project. It was reported that the induction of TK mutations is probably due to damage caused by space radiation. Notably, there was an increase in large deletion mutations [10]. It was also found that interference with DNA repair processes might result from microgravity conditions (which resulted in an ∼60% reduction in cell viability), and also that the TK mutations might be augmented by synergistic effects. Radio-adaptation was also examined after a stay of 133 days in the ISS as a possible indirect effect of space radiation [9]. Space radiation was found to enhance DSB repair mechanisms responsible for non-homologous end joining and homologous recombination. Furthermore, it appears that there was a possibility that radio-adaptation was induced when measuring the suppression of TK mutation induction (the adaptive response) [11]. These results concurred well with those observed in the Rad Gene project.

### Chromosome aberrations in *Bombyx mori* silkworm eggs induced by low-dose space radiation

PI: Toshiharu Furusawa. Heterozygous silkworm (*Bombyx mori*) eggs (*P^S^/+^p^*) were produced by a cross between a black-striped strain (*P^S^*/*P^S^*) and a normally marked strain (*+^p^*/*+^p^*). When the heterozygous strain was exposed to heavy ion particles, white spots were detected on the black dorsal surface of the larvae. The heterozygous eggs were carried to the ISS and maintained there for 91 days. The total dose equivalents received were 34.0 ± 4.3 to 41.8 ± 6.9 mSv. After returning to the Earth, no mutations occurred in the integument of the larvae hatched from these exposed eggs (the first filial generation). However, the second-generation larvae exhibited white spots on the black integument of the dorsal surface, and subsequently many white spots appeared on the backs of the smeared light black integument of the third generation *p*/*p*/*P^S^* larvae. These results suggest that space radiation affects primordial germ cells during embryonic development in the first generation.

### The effects of space radiation on mouse embryonic stem cells in the ISS

PI: Takashi Morita. Currently, samples of mouse embryonic stem (ES) cells are flown in a frozen stage to the ISS. ES cells are then inserted by micro-injection into mouse embryos, and the early development of the embryos is observed. These observations are designed to measure cell killing, chromosome aberrations, gene expression and DNA damage *in vitro* at the cellular level. The development of chimeric mice will be examined *in vivo*.

### Lifetime heritable effects of space radiation on mouse embryos after long-term storage in the ISS

PI: Shizuko Kakinuma. This experiment is aimed at clarifying the effects of space radiation in parental wild-type (B6C3F1) and DNA repair–deficient mutant mice (*Trp53*, *Scid*, *Mlh1*, *Min*, *Ptch* and *gpt-delta*). Space-X-6 was launched on 14 April 2015. These samples will return to earth ∼6 months later *via* Space-X-8. Frozen mouse embryos at the 2-cell stage were brought in a frozen state to the ISS and exposed to its radiation environment. After space flight, the eggs will be transferred into surrogate mothers and examined during their life spans. The investigators are planning to analyze animal life spans, cancer development, and tumor spectrums in latent tumors, as well as gene mutations, gene expression, chromosome aberrations, point mutations, and transgenerational DNA repair in deficient mutants and wild-type organisms.

## NEW FACILITIES PLANNED FOR THE ISS/KIBO

The JSBSS, UCSES and ACJ are supporting new experimental facilities in the ISS through 2020. JAXA's aims for basic life science studies in space are: (i) to pursue basic science research in the ISS using the best current experimental methods and analytical methods to study ‘Biological Response Mechanisms in Space Environments’; and (ii) to establish protocols and standards based on current research in the biological sciences to permit long human stays in space, and to prepare for future endeavors in space, such as trips to mars.

Another goal is to build new experimental facilities in the ISS for continuing and furthering this program. It is hoped to establish five new facilities.

### A plant culture system

New projects that are envisioned involve the culture of various types of plants for growth and cell cycle studies, and exploring the possibilities of agriculture in space for long stays in space. Of particular interest are the effects of µ*g* and 1 *g*.

### Animal breeding systems

Mammals such as mice and small rabbits are to be grown and bred in space to analyze fertilization, growth, development, movement and living under µ*g* and 1 *g* conditions. Centrifugation systems in the ISS will be used to provide different levels of gravity. Potential projects are studies of reproduction, development, birth, care of offspring, behavior and healthcare in gene knockout (mutated) and parental wild-type animals. Multichannel telemetry will be used to monitor body temperatures and behavior with a CCD camera and to download data to the Earth from the ISS.

### Studies of the localization and movement of cellular components

Gene expression, and behavior of proteins, chromosomes and organelles in the cell will be monitored with real-time analysis. Organisms will be cultured under µ*g* and 1 *g* conditions using centrifugation systems in the ISS. Microorganisms and cultured animal and plant cells will be monitored using confocal fluorescent microscopy. The aim is to obtain clear images from thin and thick samples in living cells using GFP fluorescence techniques and other methods.

### Collection of biological samples from space and their analysis

The aim is to (i) analyze samples in the ISS from space-based experiments, and (ii) to improve the collection of samples after space experiments. Currently, it is difficult to retrieve biological samples from space. Tests of the effects of vibrations and shock during landing are required because we are now unable to use space shuttles. It would be desirable to download experimental results directly to Earth from the ISS. New transfer systems scheduled for future use are the Space-X Dragon and the HTV-R. If analysis must be performed on the ISS, new systems must be developed to analyze biological samples on the ISS.

### Exposure facility at the ISS

Biological effects and precise physical dosimetry will be measured for solar radiation and space radiation by using filters to determine the UV and radiation species present, as well as low-dose/low-dose-rate exposures. Studied organisms will be small organisms, insects, small plants, and accompanying whole-body effects. It is hoped to construct a model in the space experimental facility to study chemical evolution under solar radiation to mimic the early stages of the emergence of life on earth. Test subjects will be exposed to solar particles and space radiation to observe survival, DNA damage, mutation, DNA repair, proliferation, development, morphogenesis, gene expression, protein synthesis, and protein localization and movement. Studies will be designed to make predictions for the future environment on Earth if the ozone layer is depleted.

## GOALS FOR NEW FACILITIES AND FOR SPACE SCIENCE

Future studies for proposed new facilities in the ISS are:

(i) studies of the origin of life on the Earth, adaptation to the Earth's environment, and mechanisms of evolution.
(ii) basic knowledge and techniques to promote human stays in space.
(iii) planning for healthy and long-term stays for humans in space.
(iv) protection from pollution and environmental changes on earth.
(v) education for the next generation.

These proposed projects will hopefully contribute to knowledge and to methods to enable humans to endure long-term stays in space with protection from the effects of microgravity and space radiation. Current discussions concern planning for the ISS/Kibo experiments through 2024 and post-ISS experiments.

## FUNDING

This work was supported by Grants-in-Aid (No. 25514007) for Scientific Research from the Ministry of Education, Culture, Sports, Science and Technology of Japan.
